# Dorsal Anterior Cingulate Cortices Differentially Lateralize Prediction Errors and Outcome Valence in a Decision-Making Task

**DOI:** 10.3389/fnhum.2018.00203

**Published:** 2018-05-22

**Authors:** Alexander R. Weiss, Martin J. Gillies, Marios G. Philiastides, Matthew A. Apps, Miles A. Whittington, James J. FitzGerald, Sandra G. Boccard, Tipu Z. Aziz, Alexander L. Green

**Affiliations:** ^1^Nuffield Department of Surgical Sciences, University of Oxford, Oxford, United Kingdom; ^2^Neurophysiological Pharmacology Section, National Institute of Neurological Disorders and Stroke, National Institutes of Health, Bethesda, MD, United States; ^3^Institute of Neuroscience and Psychology, University of Glasgow, Glasgow, United Kingdom; ^4^Department of Experimental Psychology, University of Oxford, Oxford, United Kingdom; ^5^Hull York Medical School, University of York, York, United Kingdom

**Keywords:** event related potentials (ERP), executive function, functional localization, intra- extradimensional set shift task, lateralization, outcome valence, prefrontal cortex (PFC), theta oscillations

## Abstract

The dorsal anterior cingulate cortex (dACC) is proposed to facilitate learning by signaling mismatches between the expected outcome of decisions and the actual outcomes in the form of prediction errors. The dACC is also proposed to discriminate outcome valence—whether a result has positive (either expected or desirable) or negative (either unexpected or undesirable) value. However, direct electrophysiological recordings from human dACC to validate these separate, but integrated, dimensions have not been previously performed. We hypothesized that local field potentials (LFPs) would reveal changes in the dACC related to prediction error and valence and used the unique opportunity offered by deep brain stimulation (DBS) surgery in the dACC of three human subjects to test this hypothesis. We used a cognitive task that involved the presentation of object pairs, a motor response, and audiovisual feedback to guide future object selection choices. The dACC displayed distinctly lateralized theta frequency (3–8 Hz) event-related potential responses—the left hemisphere dACC signaled outcome valence and prediction errors while the right hemisphere dACC was involved in prediction formation. Multivariate analyses provided evidence that the human dACC response to decision outcomes reflects two spatiotemporally distinct early and late systems that are consistent with both our lateralized electrophysiological results and the involvement of the theta frequency oscillatory activity in dACC cognitive processing. Further findings suggested that dACC does not respond to other phases of action-outcome-feedback tasks such as the motor response which supports the notion that dACC primarily signals information that is crucial for behavioral monitoring and not for motor control.

## Introduction

The dorsal anterior cingulate cortex (dACC), located in the medial prefrontal cortex (PFC), has been associated with a broad range of executive functions including salience (Seeley et al., 2007), conflict monitoring (Botvinick et al., 2001; Botvinick, 2007), error detection (Holroyd and Coles, 2002; Ito et al., 2003; Hyman et al., 2013), and reward-based decision making (Walton et al., 2003; Behrens et al., 2007; Kolling et al., 2016a). A prominent theory of dACC function suggests the dACC monitors both external and internal environments, makes predictions, observes outcomes, and provides a summary report of outcomes to downstream circuits (Schall et al., 2002; Heilbronner and Hayden, 2016). While dACC monitoring signals are usually observed after decisions and feedback, in some cases dACC signaling can occur throughout the decision-making process allowing for real-time updating of performance (Carter et al., 1998; Holroyd and Coles, 2002; Blanchard and Hayden, 2014). Likewise, while it is universally accepted that dACC neurons are sensitive to error commission, as evidenced by error-related negativity signals in event-related potential (ERP) studies, the strict view of the dACC as exclusively an error detector has been generally rejected (Amiez et al., 2005). Most likely, the dACC reacts to error as one of a series of stimuli that drive the region. For example, the dACC has been shown to increase in activity in contexts where errors are likely but do not actually occur (Brown and Braver, 2005). Conflict monitoring, on the other hand, proposes that ongoing levels of conflict or competition are tracked by the dACC and signaled as additional cognitive resources are required (Botvinick et al., 2001). While this is an appealing idea often suggested in neuroimaging studies, there is scant supporting evidence in electrophysiological recordings (Nakamura et al., 2005; Cai and Padoa-Schioppa, 2012; Sheth et al., 2012).

As a result, much of the research into dACC function has attempted to propose generic, computational models that unify dACC functions focused on the vital role dACC plays in learning. The various models based on single-unit recording and local field potential (LFP) recordings in non-human primates converge to propose that neurons in the dACC signal predictions of some parameter, whether external or internal, that range from the volatility of the reward environment to the optimal value of cognitive control (Behrens et al., 2007; Alexander and Brown, 2011; Shenhav et al., 2013; Silvetti et al., 2014). These predictions act to encode one’s expectations for the likely outcomes of decisions or actions (Hayden et al., 2011; Kennerley et al., 2011; Cai and Padoa-Schioppa, 2012; Procyk et al., 2016). After feedback, these dACC neurons signal both the valence of the outcomes of one’s behavior—either positive or negative—and further respond to valence via prediction error signaling on axes such as good/bad and expected/unexpected (Philiastides et al., 2010; Hayden et al., 2011; Guitart-Masip et al., 2014). A positive valence is associated with actions that result in a reward (e.g., a successful or desirable outcome) while negative valence is associated with an undesirable outcome. Such activity is used to update predictions and optimize future behavior. Ultimately, questions remain regarding dACC function. Are these functions performed by anatomically discrete subregions? Is dACC function lateralized across hemispheres? How does the dACC, especially in the human PFC, perform such processes? This last question requires intracortical electrophysiology which is extremely rare in humans as there are few clinical justifications to warrant placing recording electrodes in or near the dACC. In light of the many theories of dACC function, we hypothesize that the dACC maintains predictive information about the outcomes of one’s behaviors, monitors the valence of behavioral outcomes, and uses valence and resulting prediction errors to drive behavioral adaptations. Further, we hypothesize that these executive functions in humans are a result of underlying electrophysiology in the form of or reflected by LFPs as is observed in non-human primates.

**Figure 1:**
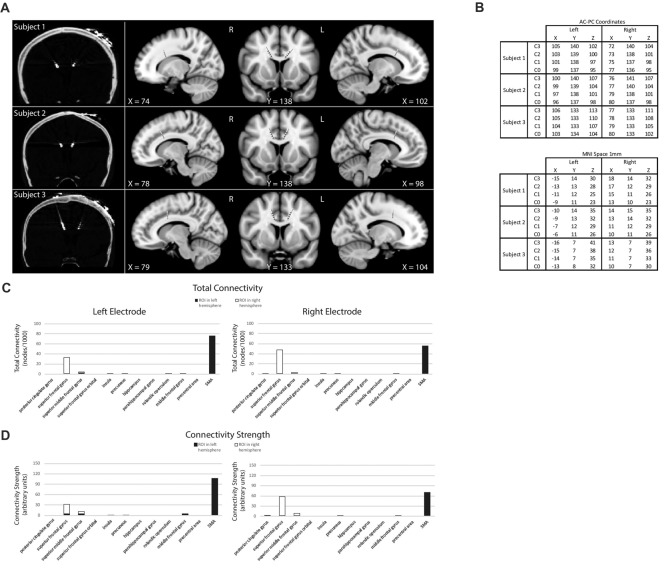
Subject electrode placement **(A,B)** and diffusor tensor imaging (DTI) data **(C,D)**. **(A)** Post-operative computed tomography (CT)-scans showing subjects’ electrode placements. Electrodes registered and displayed in the common Montreal Neurological Institute (MNI) space. **(B)** Electrode contacts in AC-PC coordinates and MNI space. Electrode order from most dorsal, C3, to most ventral, C0. **(C)** DTI-computed total connectivity derived from the number of voxels with non-zero connectivity with several regions of interest (ROI) available from two of three subjects. DTI connectivity is from the middle electrode contact pair C2-C1. Both left and right electrodes displayed connectivity to right superior frontal gyrus (SFG) and left supplementary motor areas (SMA). **(D)** DTI connectivity strength as mean intensity per non-zero voxels. Supporting total connectivity results, highest connectivity strength was with right SFG and left SMA.

The introduction of deep brain stimulation (DBS) to treat certain neurological disorders has allowed for some of these questions to be explored in humans through the recording of electrophysiology while subjects perform behavioral tasks. To test our hypotheses, we recorded LFPs from the dACC bilaterally, in three habitually right-handed subjects undergoing DBS for chronic pain, allowing the precise examination of whether prediction signals (reactions pre-feedback to stimuli presentation), outcome valence signals (reactions post-feedback to the intrinsic attractiveness or averseness of an outcome), prediction error signals (reactions post-feedback to differences between predicted and eventual outcome) are localized to the dACC (Figures 1A,B). By recording bipolar mode LFP, we were able to precisely localize LFP to the dACC, take recordings from within a few millimeters of the electrodes, minimize volume conduction effects from distant areas, and record simultaneously from both hemispheres of the dACC (Lempka and McIntyre, 2013). Diffusor tensor imaging (DTI) was performed postoperatively to confirm DBS electrode placement and to ensure the bilateral contacts were capturing electrophysiological signals from comparable, symmetrical regions in the dACC.

Participants performed a modified Wisconsin card-sorting cognitive test (Intra- Extradimensional Set Shifting test, IED). IED is a measure of attentional set shifting, assessing cognitive flexibility and executive function in one’s ability to switch between arbitrary internal rules (Keeler and Robbins, 2011; Scheggia et al., 2014). Its physical variation, Wisconsin Card Sorting, is the most widely used neuropsychological task for the evaluation of this function in humans (Eling et al., 2008; Barnett et al., 2010). IED has been used to identify executive function abnormalities in a wide range of mental disorders including attentional deficit disorders, obsessive-compulsive disorders, and Parkinson’s disease (Head et al., 1989; Owen et al., 1993; Chamberlain et al., 2011). Attentional set shifting tasks allow for the selective measurement of the processes underlying discriminative learning, reversal learning behavior, and the switching of attention within both the same dimension (during intradimensional shifts) and an alternate dimension (extradimensional shifts) in a tested subject. Such a distinction is relevant, as functional specialization within the PFC has been observed to govern these two types of shifts. This has been demonstrated between the orbital regions and the lateral (in non-human primates) and medial (in rodents) regions in the PFC, respectively (Scheggia et al., 2014). Orbitofrontal cortex has been shown to be selectively involved in reversal shifts, while the lateral/medial PFC has been shown to be involved in the extradimensional shift (Dias et al., 1996; Hampshire and Owen, 2006; Keeler and Robbins, 2011).

**Figure 2:**
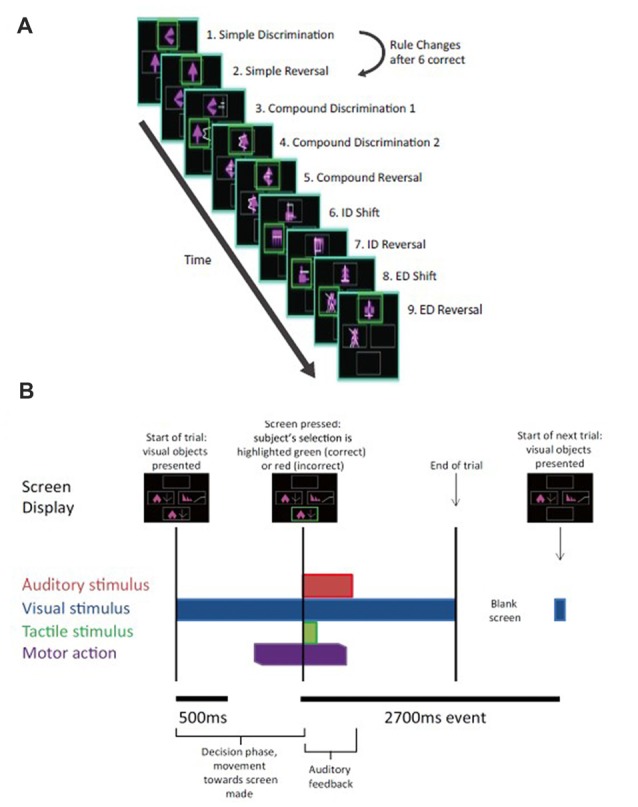
Intra- extradimensional set shift task (IED).** (A)** Schematic of the IED task from the Cambridge Neurophysiological Test Automated Battery (CANTAB) displaying rule order during a given recording. Green rectangles indicate the correct choice. The rule progresses after six consecutive trials with correct responses. The test terminates if six consecutive correct trials cannot be made over a period of 50 attempts. Copyright 2008 Cambridge Cognition, Ltd. All rights reserved. **(B)** Schematic representation of IED sensory and motor events within a given trial. A trial begins with the presentation of two visual, abstract objects. After a variable-length decision-making phase, the subject then makes a movement to touch the CANTAB test screen with their dominant hand. A screen press elicits auditory and visual feedback indicating whether the subject has chosen the correct or incorrect figure for the current rule. After an interval of 1.5 s, the screen turns blank and then begins the next trial. Reproduced with permission from Figure 1 of Gillies et al. (2017), under the open access Creative Commons Attribution 4.0 International License (http://creativecommons.org/licenses/by/4.0/).

On each IED trial, the subject chooses between pairs of stimuli—with one stimulus a “correct” match to target and the other an “incorrect” match to target, based on a rule unknown to the subject—with success or failure indicated through auditory feedback following their choice (Figure 2). Once the rule defining the correct stimulus-outcome had been learned—as evidenced by several consecutive correct trials in a row—unexpected rule changes occurred leading to discrepancies between the predicted and actual validity of a subject’s choice. In addition to unexpected rule changes, the target visual stimuli presented also changed from trial to trial, resulting in the presentation of familiar and novel stimulus pairs. In brief, subjects are presented with two stimuli, they make a choice, an outcome is signaled, and this pattern is repeated which informs future rounds. As rule changes occur unpredictably once a rule is learned by a subject, the IED task has elements of learning (e.g., is the rule X or Y?), prediction (e.g., the rule in the previous trial was X and so it should still be X), and an outcome that can be manipulated to be expected or unexpected (e.g., the rule was X as expected or was unexpectedly changed to Y). Using this design, we searched for electrophysiological correlates of predictive activity at the time that pairs of stimuli were presented and outcome valence or other error-related activity at the time of outcome feedback in left and right dACC, contributing unique electrophysiological information to the study of dACC function not obtainable through imaging or directly via surface electrodes in humans.

## Materials and Methods

### Subject Group and Ethics Approval

Three subjects suffering from chronic pain (one female, two male, average age = 42 years, standard deviation = 4.9 years) were studied. All three subjects were habitually right-handed (Table 1). This study was carried out in accordance with the recommendations and approval of Oxfordshire Research Ethics Committee A (Ref 11/SC/0229). All subjects gave written informed consent in accordance with the Declaration of Helsinki.

**Table 1:** Subject data.

Subject	Age (years)	Sex	Handedness
1	48	M	Right
2	36	F	Right
3	42	M	Right

*The subject group is described in Table 1. Three subjects (one female and two male) with chronic pain were studied. The average age of subjects at the time of surgery was 42 years, with a standard deviation of 4.9 years. All three subjects were habitually right-handed*.

### Surgery

All three subjects underwent bilateral dACC DBS surgery. dACC targets were selected on preoperative magnetic resonance imaging (MRI) scans. Selected targets were 20 mm posterior to the frontal horns and 8–10 mm lateral to the midline to target the dACC. The tip of each electrode was targeted to contact the corpus callosum such that as many contacts lay within the cingulate bundle as possible. Subjects underwent general anesthesia and Cartesian coordinates were generated for preselected targets using a combination of Brown-Roberts-Wells stereotactic localizer frames, preoperative computed tomography (CT) head scans performed under anesthesia and NeuroInspire^®^ (Renishaw plc, Wotton-under-edge, UK) image fusion software. Cartesian coordinates were then configured on the Cosman-Roberts-Wells frame attached to the subject’s head. A 2.7 mm twist drill craniostomy was made and Medtronic 3387 DBS leads were passed to target coordinates, with extension leads attached and externalized. Each DBS lead has four circumferential 1.5 mm electrodes separated by 1.5 mm. A second CT head scan was performed to check lead position before recovery from anesthesia. Internalization of DBS leads and implantation of internal pulse generators took place a week later after clinical testing for efficacy.

### MRI Acquisition

Before DBS surgery, subjects underwent a T1- and T2-weighted MRI scan on a Philips Achieva 1.5 Tesla magnet. Diffusion-weighted data were acquired using a single-shot echo planar sequence. The scanning parameters were as follows: echo time, 65 ms; repetition time, 9390 ms; 176 × 176 reconstructed matrix; voxel size of 1.8 × 1.8 × 2 mm; and slice thickness of 2 mm.

### DTI Processing

DTI data were acquired with 33 optimal nonlinear diffusion gradient directions, 1200 s/mm^2^, and one non-diffusion-weighted volume, 0 s/mm^2^. DTI pre-processing was performed using the Oxford Centre for Functional MRI of the Brain Software Library (FSL) tools comprising eddy current and head motion correction, brain extraction, diffusion tensor fitting on correct 4D-data and modeling of crossing fibers using an automatic estimation of 2-fiber orientations per voxel (Smith et al., 2004; Woolrich et al., 2009; Jenkinson et al., 2012). The electrode contact coordinates were determined as described in a previous tractography study (Boccard et al., 2016). For the present work, DTI scans were available for two out of the three subjects. As the LFP were recorded between two adjacent electrode contacts, we defined as seed each couple of adjacent contacts: the most ventral two as C0 and C1, the middle two as C1 and C2, and the most distal two as C2 and C3. For each subject, the connectivity was computed between each of these seeds and brain areas of interest. For both brain hemispheres, we measured the connectivity patterns to several areas of the automated anatomic labeling template in the 2 mm Montreal Neurological Institute (MNI) space: the hippocampus, the insula, the middle frontal gyrus (MFG), the posterior cingulate gyrus (PCG), the precuneus, the Rolandic operculum, the superior frontal gyrus (SFG), the superior middle frontal gyrus (SMFG) and the supplementary motor area (SMA). For each subject, we ran probabilistic tractography using the C0–1, C1–2 and C2–3 electrode seed areas in each subject’s DTI space. Five thousand sample streamlines were seeded from each voxel of the seed region. The probabilistic tractographies obtained were subsequently registered to the 2 mm MNI space. We then computed the total connectivity, the number of voxels with a non-zero connectivity, and the connectivity strength represented as mean intensity per non-zero voxels within the masks of the above brain areas.

### Intra- Extradimensional Set Shifting Task

Subjects performed an on-screen variation of the Wisconsin Card Sorting Test called the Intra- Extradimensional (IED) Set Shifting task (Cantab^®^). Subjects learn a series of nine two-alternative forced-choice discrimination rules between two visual objects presented on-screen (Figure 2A). A script is read to the subject before the test begins, informing them to pick one of two on-screen visual objects. They are informed one object is “correct” and the other “incorrect.” They are informed there is no stimulus characteristic which indicates which object is correct or incorrect on the first trial, but that the computer gives feedback after selection to inform them whether they selected the “correct” or “incorrect” object. Subjects are informed that the rules will change over the course of the test, but that these rules do not change often, and rule changes occur only once the preceding rule has been learned. They are not informed how many times the rules will change or how many correct trials in a sequence they must achieve before the rule changes. Selection of an object on-screen by touch causes an audible tone (pure tone for correct, low frequency modulated pure tone for incorrect) and simultaneous presentation of a colored box around the edge of the screen (green for correct and red for incorrect) with the word “correct” or “incorrect” lasting 0.5 s, which indicates whether the choice was correct or incorrect (Figure 2B). The next trial begins automatically 2.7 s after the start of the previous trial’s object selection auditory feedback. Subjects were invited to use their dominant hand for object selection (in all three subjects the right hand). The rule is defined as “learned” when the subject achieves six correct choices in a row, prompting progression to the next rule. At the start of the test, each of the two visual objects are composed of a single solid abstract shape (the internal dimension), which may occupy one of four on-screen rubrics. The spatial relationship between the two visual objects varies randomly as a distractor during a sequence of trials governed by the same rule. Once the subject has learned rule 1 (i.e., which is the correct solid shape), the computer switches the rule (simple reversal) and the subject (having been trained or manipulated into predicting or “expecting” rule 1) receives “unexpected” incorrect feedback and now must learn that an alternative shape is now the “correct” one (step 2 in Figure 2A). As the task progresses, the visual objects acquire an additional abstract element in the form of white lines (the external dimension). These white line figures (e.g., rule 3 in Figure 2A) act as distractors during rules 3–5 (compound discrimination 1, compound discrimination 2 and reversal), since the rule is based on the solid shape component of the objects only. During sequences governed by rules 3–5, the white line figures randomly associate with each solid shape, with a small variable range of geometric relationships to the solid component. Upon transition to rule 6 (intradimensional shift), the solid shapes change to two new visual objects composed of new solid shape and white line figures, but the rule governing correct object choice is determined by the solid shape component only (i.e., intradimensional component), not the white line figures. The white line figure acts as the stimulus dimension governing correct object choice during the extradimensional phase of the task (rules 8, extradimensional shift, and 9, reversal). During transition to reversal rule trials (rules 2, 5, 7, 9) the visual objects do not change compared to the last trial of the previous rule. During transition to discrimination and dimensional shift rule trials (rules 3, 4, 6, 8) the on-screen visual objects change (although some component elements of the objects may not) compared to the last trial of the previous rule. The subject passes the task if he/she learns all nine rules in sequence. The subject fails if he/she does not achieve six correct trials in sequence out of 50 trials of a single rule.

### Electrophysiology and Analysis

Differential recordings were made from adjacent circumferential 1.5 mm contacts of each deep brain macroelectrode in a bipolar configuration to limit the effects of volume conduction and limit spatial resolution of recordings to a few millimeters of adjacent tissue (Parra et al., 2005). dACC contacts were identified by postoperative image-fused MRI and CT. Signals were high-pass filtered at 0.5 Hz, amplified (10,000×) and digitized at a rate of 2.5 kHz using a Porti system (Twente Medical Systems international, B.V., Netherlands) and recorded onto disc using Spike2 software (Cambridge Electronic Designs, Cambridge, UK). Raw data were notch filtered at 50 Hz, 100 Hz and 150 Hz as required using Spike2 infinite impulse response Bessel filters, Q value adjusted to minimize unwanted filtering of adjacent frequencies. Pre-processing and analysis of LFPs were performed offline using MATLAB software (Mathworks Inc., Natick, MA, USA) and EEGlab (Delorme and Makeig, 2004; Delorme et al., 2011). Recordings were taken from awake, behaving subjects at room temperature.

Spike2 data were imported into EEGlab. Raw data were resampled at 512 Hz. Six-second epochs (beginning −4000 ms prior to the start of auditory feedback continuing to +2000 ms) were extracted from left and right dACC contacts and divided into correct and incorrect trials as appropriate. Trials were sub-divided into simple correct trials and incorrect trials and further into correct and incorrect trials that were expected and unexpected. An expected correct trial was one in which the previous five trials had received correct feedback. An unexpected correct was derived from a first correct response to novel stimuli—a guess. An expected incorrect was the first incorrect response to novel stimuli—a guess. An unexpected incorrect was obtained from the first incorrect trial of a reversal rule set. Baseline prior to feedback (−2000 ms to 0 ms) was subtracted, then data were normalized by individual mean and sample standard deviation using MATLAB z-score command to allow comparison between different subjects. EEGlab commands were used to generate ERP, power spectra and event-related spectral perturbations (ESRP). ESRP is a form of wavelet-based time-frequency analysis that measures average dynamic changes in the spectral amplitude relative to an experimental event common baseline, to compare responses in the range of 3–100 Hz (Duda et al., 2001). The time between the presentation of stimuli on the IED test screen and the subject touching the screen to make their selection was recorded as a trial’s reaction time (RT).

### Multivariate LFP Discriminant Analysis

We applied a linear multivariate classifier to LFP data locked to the time of feedback to discriminate between positive vs. negative decision outcomes using a sliding window approach. Only results from subject 1 and subject 2 were used, as subject 3 did not produce enough incorrect trials to reliably train the multivariate discriminant. Specifically, we estimated a projection of the multidimensional LFP signals, ***x***_i_(t), where *i* = {1…*T*} and *T* is the total number of trials, within a short time window, τ, that maximally discriminated between positive and negative outcome trials. Each time window had a width of *N* = 50 ms and the window center was shifted from −200 ms to 600 ms relative to outcome onset, in 10 ms increments. We used logistic regression to calculate the spatial weighting, ***w***(τ), that achieved maximal discrimination between positive and negative outcomes, arriving at the one-dimensional projection *y*_i_(τ), for each trial *i* and a given window τ:

(1) $$y_{i}(\tau )=\frac{1}{N}\sum_{t=\tau -N/2}^{t=\tau +N/2}w{\left(\tau \right)}^{⊥}x_{i}(\text{t})\;$$

where ⊥ is used to indicate a transpose operator (Parra et al., 2005). Note that the classifier was designed to map positive and negative discriminant component amplitudes (i.e., *y*_i_(τ)) to positive and negative outcomes, respectively.

We quantified the performance of the discriminator for each time window using the area under a receiver operating characteristic curve, referred to as an *A_z_* value, using a leave-one-out trial cross-validation procedure (Parra et al., 2005). We utilized a bootstrapping technique to assess the significance of the discriminator to a significance level of *P* < 0.01.

To visualize the temporal profile of the resultant discriminating components, we applied the spatial weighting vectors, ***w***(τ), from the short time windows, that led to significant discrimination performance between positive vs. negative outcomes, to an extended time window (from 200 ms before until 600 ms after the outcome).

### Experimental Design and Statistical Analysis

In this study, we aimed to test the electrophysiological response of the human dACC to a stimuli-feedback-response cognitive task using the unique opportunity of externalized DBS patients. We did not perform prospective sample size calculations as post-operative recordings from DBS electrodes—particularly in the PFC—are a rare research opportunity. Thankfully, as bipolar LFP recordings have a relatively high signal-to-noise ratio and minimize volume conduction effects from without, a small number of subjects (as low as two) with a large trial count is considered sufficient to detect effects (e.g., Womelsdorf et al., 2010). To that extent, we reported the total number of given trial types—correct and incorrect trials, and familiar and novel stimuli trials. To properly isolate sources of variation in measurements to improve statistical testing, additional biological (rather than technical) replicate measurements were utilized. Our biological replicates, as defined by Blainey et al. (2014) as “parallel measurements of biologically distinct samples that capture random biological variation, are the recordings made from two of the subjects we analyzed. Of the three recorded subjects, subject 3 had to be excluded from the multivariate discriminant analysis as he did not produce enough incorrect trial data to reliably train the multivariate discriminant as he did not produce enough incorrect trial data to reliably train the multivariate discriminant.

Across all subjects, we collected a repeated number of technical replicates of 796 total trials, broken down into 568 correct trials and 228 incorrect trials, and further into 66 trials with familiar stimulus pairs and 33 trials with novel stimulus pairs. Exact *p*-values for the cluster-based permutation correction procedure we used varied in each run due to the nature of random permutations, and thus were not reported. EEGlab non-parametric permutation statistics with false discovery rate (FDR) correction were used to compare LFP data between trials and between study groups. To assess the significance of the multivariate LFP discriminator, we used a bootstrapping technique where we performed the leave-one-out test after randomizing the trial labels. We repeated this randomization procedure 1000 times to produce a probability distribution for *Az*, and estimated the *Az* leading to a significance level of *P* < 0.01. Non-parametric Rank Sum and Kruskal-Wallis tests were used to analyze RT as RT data were not normally distributed. RT data are therefore expressed as medians within the 25%–75% interquartile range.

## Results

Three electrode contact pairs at different coordinates were available for use in recording (Figure 1B). The central pair, C2–1, was chosen to orient recordings anatomically directly within the dACC. MRI DTI was performed to assess the orientation and integrity of white matter tracts between the electrode position and multiple regions of interest (ROI) to confirm electrode positions. This analysis was available for two of the three subjects (Figures 1C,D). For both subjects and both electrodes, the strongest connectivity was found to be the left hemisphere’s SMA. A notable connectivity was also found to the right hemisphere’s SFG and the SMFG.

A total of 796 IED trials were available for analysis, and subject data were pooled for respective hemispheres, stimulus type, and feedback valence—whether the subject made a correct or incorrect choice. Subjects made a total of 568 choices resulting in correct feedback and 228 choices resulting in incorrect feedback (Table 2). Additionally, trials were sorted according to trends—whether trials resulting in either correct or incorrect feedback were followed by a subsequent trial resulting in correct or incorrect feedback—in order to observe behavioral changes. Subjects made consecutive correct trials in 76.3% of cases and made consecutive incorrect trials in 40.6% of cases. Detailed RT data were available from all three subjects. When considering RT, there was no significant difference (*P* > 0.40) between the average correct trial (median RT: 938.5 ms, interquartile range: 709–1372.7 ms) and the average incorrect trial (median RT: 923.5 ms, interquartile range: 722.5–1459.3 ms). Likewise, there were no significant differences in RT between trials when sorted by trend as shown in Table 2, with the exception of the RT of consecutive correct and consecutive incorrect trials. Subjects performed the second of consecutive correct trials (median RT: 911 ms, interquartile range: 701–1332 ms) significantly faster (*P* < 0.05) than the second of consecutive incorrect trials (median RT: 1016 ms, interquartile range: 731.75–1780 ms).

**Table 2:** Intra- Extradimensional (IED) results.

Correct trials (*n* = 568)		Incorrect trials (*n* = 228)	
… following incorrect trial	… following correct trial	… following incorrect trial	… following correct trial
137	431	94	134

*A total of 796 trials were available over three subjects. Five-hundred and sixty-eight trials resulted in correct feedback and 228 trials resulted in incorrect feedback. Trials (whether correct or incorrect) were further sorted into whether they were proceeded by a correct or incorrect trial to gauge behavioral changes. 76.3% of correct trials were followed by a succeeding correct trial. 40.7% of incorrect trials were followed by a succeeding incorrect trial*.

**Figure 3:**
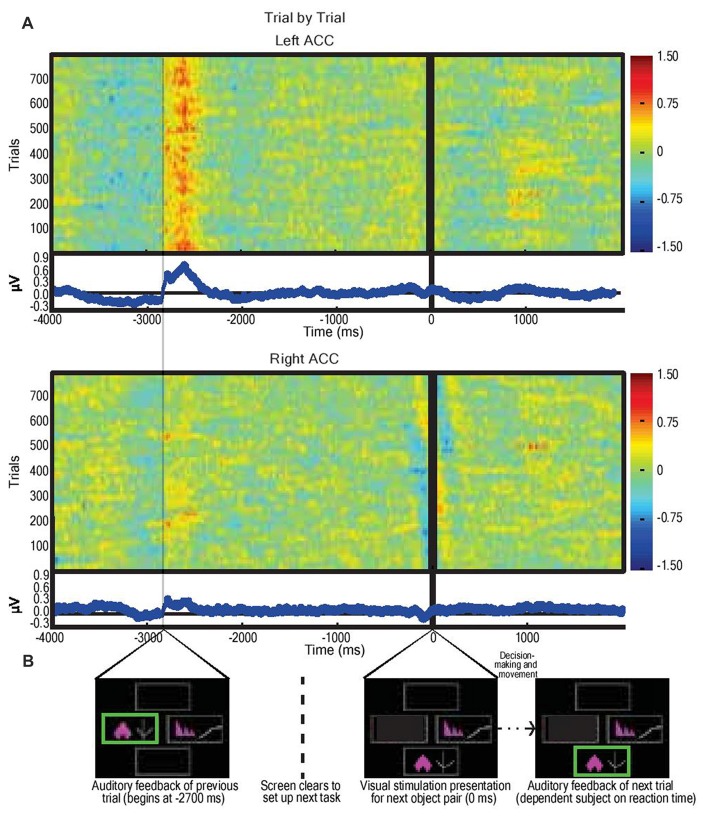
Average dorsal anterior cingulate cortex (dACC) bipolar mode field potential response in three subjects performing IED task, trial by trial responses. **(A)** Trial-by-trial field potential responses (y axis, *n* = 796 trials, three subjects) from left and right dACC (regardless of correct or incorrect result) vs. time representing the decision-making period composed of: selection of object (−4000 ms to −2700 ms); receipt of feedback (−2700 ms to −2100 ms); clearing of the screen (−1500 ms to −200 ms); and object pair presentation of the subsequent trial (0 ms onwards). Color represents magnitude of the field potential (red = higher voltage) such that individual pixels represent the magnitude of the local field potential (LFP) at a point in time in a trial. The trials were locked to stimulus presentation (0 ms). The time delay between start of feedback of the preceding trial and presentation of visual object pair in the next trial was constant (2700 ms). Responses were normalized (x-mean standard deviation) but not filtered. Blue line graphs represent average of individual trial responses with Y-axis representing normalized voltage and X-axis representing time. The most notable result is the evidence of a left dACC event related potentials (ERP) response to feedback at approximately −2700 ms with a magnitude of 0.8 μV. A lesser but still significant (*P* < 0.05) response also appeared in right dACC at this time with a magnitude of 0.3 μV. There was no apparent response to visual object presentation (0 ms) nor to movement (prior to −2700 ms). **(B)** Sample IED images aligned with events from Figure 2B.

LFPs from left and right dACC were averaged at time of stimulus presentation across all trials incorporating visual object presentation, motor action, and feedback phases (Figure 3). The most prominent feature of the averaged response, consistent across trials and subjects, was that feedback was associated with an ERP beginning 50 ms after the start of feedback in the left hemisphere with a mean peak magnitude of 0.8 μV, more prominently than the right hemisphere with a peak of 0.3 μV (*P* < 0.05; Figure 3A).

**Figure 4:**
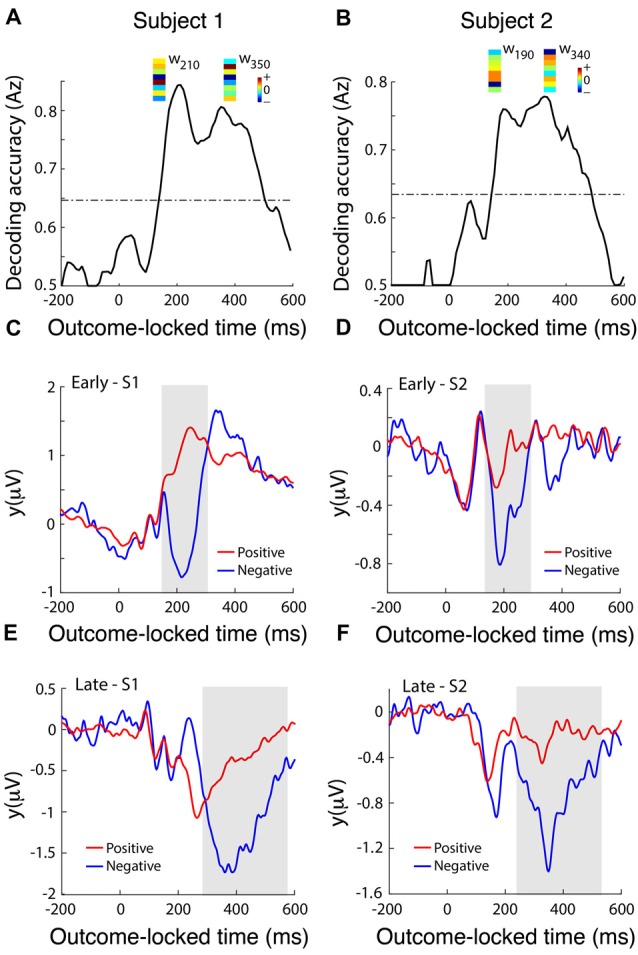
dACC discriminates positive vs. negative feedback. **(A,B)** Decoding performance (Az) during outcome valence (positive-vs-negative outcome) discrimination of feedback-locked monopolar LFP data for subject 1 **(A)** and subject 2 **(B)**. Subject 3 did not produce enough incorrect trials to reliably train the multivariate discriminant. The dashed line represents the subject-specific *Az* value leading to a significance level of *P* = 0.01, estimated using a bootstrap test. Spatial weights (*w*) of subject-specific discriminating (early and late) components are shown over the relevant peak component times. These weights represent the relative contribution of each LFP electrode to the overall discrimination performance (the sign of the weights is arbitrary and depends on the polarity of the corresponding electrode signals). Reproduced with permission from Figure 1 of Gillies et al. (2017), under theopen access Creative Commons Attribution 4.0 International License (http://creativecommons.org/licenses/by/4.0/). **(C,D)** Temporal profile of the early discriminating component activity (*y*(*early*)) averaged over trials (for subject 1 and subject 2 shown in **A** and **B**, respectively) for each of the positive (red lines) and negative (blue lines) outcomes, obtained by applying the subject-specific spatial weights estimated at the time of maximum discrimination (see timing of *w*’s shown in **A,B**) over an extended time window spanning the delivery of feedback (−200 ms to 600 ms post-feedback). The gray shaded area is used to highlight the range over which the difference between the two outcome types is more prominent. **(E,F)** The temporal profile of the late discriminating component activity (*y*(*late*)) for each of the positive and negative outcomes. Same convention as in **(C,D)**.

To identify temporally distinct neuronal population components associated with the value of outcome, we used single-trial multivariate discriminant analysis on LFP signals locked to the delivery of feedback to extract information on the prediction valence generated by the dACC (Lempka and McIntyre, 2013). We analyzed this post-feedback ERP in more detail by running a multivariate discriminant analysis on the broadband signal to integrate information across DBS electrodes and generate an aggregate discriminator channel that best dichotomized outcomes into positive (correct) and negative (incorrect) outcomes (Fouragnan et al., 2015, 2017). Discrimination performance increased in the range 200–400 ms following the outcome, with two distinct temporal components peaking roughly at 200 ms (early) and 350 ms (late) corresponding to 6.6 Hz or one theta frequency oscillation (3–8 Hz) period apart (Figures 4A,B). Using a univariate discrimination—by considering individual LFP channels in isolation—was consistently less reliable. Similarly, by comparing dACC subregions through examining spatially separated electrode contacts, the spatial weights discriminating outcome valence (***w*** in Equation 1) were only moderately correlated between the two components (Figures 4A,B), suggesting that different sub-groups of neurons within the dACC, or some other degree of spatial or lateral specialization, might be responsible for the early and late discriminating activity. Next, we computed the temporal profiles of the early (Figures 4C,D) and late (Figures 4E,F) components (***y*** in Equation 1) for each subject by subjecting the outcome-locked data through the spatial generators (weights) estimated at the peak times of the two components. These temporal profiles were highly consistent across the participants and revealed that both the early and late outcome components appear to be driven primarily by negative outcomes and the early component appears to represent a more transient event compared to the late component, which exhibited a broader response profile.

**Figure 5:**
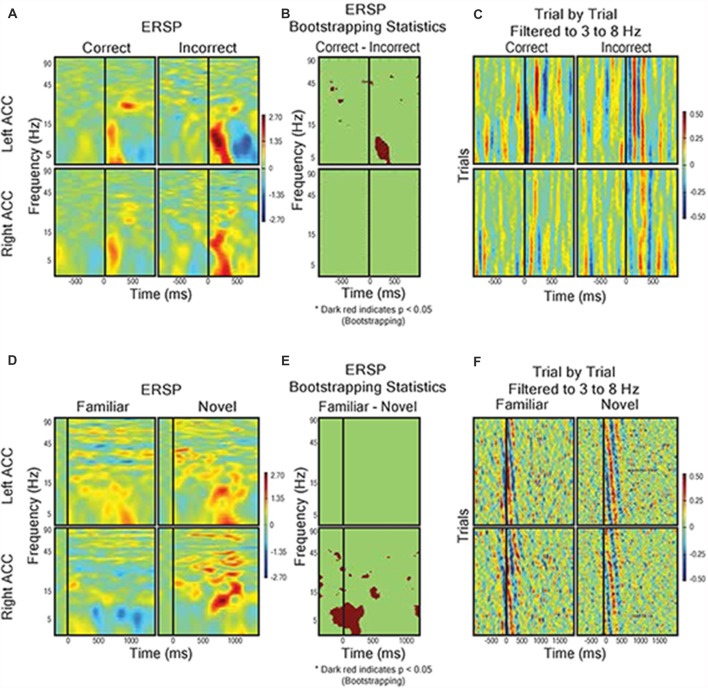
Field response to feedback **(A–C)** and visual object presentation **(D–F)**.** (A)** Correct trials (*n* = 568) vs. incorrect trials (*n* = 228). Graph shows event related spectral perturbance (ERSP) using EEGlab morlet wavelet-based analysis. Start of feedback = 0 ms and Y axis = log-based frequency (3–100 Hz). **(B)** Bootstrapping (*p* < 0.05) statistical comparison of difference in ERSP between correct and incorrect, showing frequency and time points of statistical significance. **(C)** Trial by trial LFP responses from left and right dACC during correct trials and incorrect trials filtered to theta frequency (3–8 Hz). The trials were averaged to receipt of feedback (0 ms). Color represents magnitude of the LFP (red = higher voltage) such that individual pixels represent the magnitude of the LFP at a point in time in a trial. **(D)** ERSP from left and right dACC in response to familiar stimulus pairs (i.e., stimulus of a sequence of six correct responses, *n* = 66) and novel stimulus pairs (i.e., first trials of two new object pairs, *n* = 33). Stimulus onset = 0 ms and Y-Axis as in **(A,B)**. We found a significantly greater theta frequency response of right dACC to novel stimuli but no significant differences in left dACC. **(E)** Bootstrapping (*p* < 0.05) statistical comparison of difference in ERSP between familiar and novel stimuli presentation. **(F)** Trial-by-trial data for visual object presentation filtered to theta frequency.

Given the prominence of the outcome-related activity in the dACC, we analyzed the LFP response to feedback in more detail using ERSP analysis (Makeig, 1993). We hypothesized that, supposing the dACC is involved in executive function both before and after feedback, we would detect electrophysiological activity related to novel vs. familiar objects (pre-feedback) and expected vs. unexpected outcomes (post-feedback). We compared trials with correct predictions and incorrect predictions without regard to the underlying trial rule (Figures 5A–C). The most prominent feature of the ERSP was that incorrect prediction was associated with a significantly greater response in the theta frequency band (3–8 Hz) than correct prediction in the dACC of the left hemisphere (bootstrapping with FDR, *P* < 0.05). We found no difference in outcome-related theta frequency activity between correct and incorrect trials in the dACC of the right hemisphere (*P* > 0.05; Figures 5A–C). In contrast, during stimulus presentation, there were no significant differences between ERSP during presentation of novel stimuli and familiar stimuli in the left dACC, but the right dACC displayed significantly greater ERSP to novel stimuli than to familiar stimuli in the theta frequency band (Figures 5D–F).

## Discussion

### Outcome Valence and Prediction Errors

Considerable evidence from neurophysiological recordings in non-human primates supports the claim that the dACC signals crucial information resulting from unexpected outcomes that guide learning behavior (Amiez et al., 2005; Sallet et al., 2007; Kennerley et al., 2011). Unexpected outcomes are crucial for learning; they signal the need for updating expectations about the environment. While neuroimaging studies in humans have generally supported this, their low levels of spatial resolution have failed to localize these signals to different, distinct regions within either the dACC or adjacent regions of the medial PFC (Yeung et al., 2004). This study is one of only a limited collection of studies to examine responses from direct electrophysiological recordings of human dACC during a cognitive control task. We hypothesized that electrophysiology in the form of LFPs would localize to the dACC both prediction errors and outcome valence at the time of decision feedback. We utilized a cognitive task that involved presentation of object pairs, a motor response, and audiovisual feedback to guide future object selection choices, which allowed for the manipulation of expectancy through stimulus familiarity and permitted us to probe both prediction error and valence event-related electrophysiology. Our most marked finding was that the dACC signals when the outcomes of decisions were unexpectedly incorrect. We provide strong support that the human dACC signals both prediction errors and valence. We do not see particularly striking dACC activity during movement phases of the task, suggesting that dACC signals information that is crucial for monitoring our behavior rather than for actions themselves. This aligns with dACC models that include a large role in motor control, such as that proposed by Holroyd and Coles, and findings from both human and animal studies that generally propose the dACC plays a role in selecting and maintain action policies (Holroyd and Coles, 2002; Holroyd and Yeung, 2012; Holroyd and McClure, 2015; Procyk et al., 2016; Shahnazian and Holroyd, 2018). While there is no obvious motor-related activity in-between stimulus presentation and feedback receipt as expected (at least in the left hemisphere dACC), IED as a task is not particularly suited to probe questions of motor control as it is limited to a consistent motor behavior (arm reaches) that do not vary task-to-task.

What role do prediction error signals in the dACC serve? Current computational models of the dACC highlight the dACC’s role in learning. Both the Prediction Response Outcome model of Alexander and Brown and the reward value and prediction model of Silvetti et al. (2014) propose that the dACC signals prediction errors resulting from unexpected outcomes (Alexander and Brown, 2011). Computational models have also suggested that dACC signals not only reflect how surprising an outcome might be, but are also crucial for learning and updating predictions after feedback and driving subsequent adaptive changes to behavior (O’Reilly et al., 2013). Indeed, this could be seen in the data. Not only were subjects more likely to make consecutive correct choices and thus repeat successful behavior; but they were more likely to follow an incorrect trial (with its associated higher dACC activity) with a subsequent correct trial and therefore improve their behavior (Table 2). This implies that the selection of actions, and potentially the speed with which actions are made, are influenced by the valence (the “correctness” or “desirability”) of one’s predicted outcomes (Guitart-Masip et al., 2014). While there is modest evidence of post-error slowness in our RT results, we cannot meaningfully divide our RT data by rule-type and thus we cannot say how RT relates to dACC activity. Our results provide support for these models of dACC function and for the first time demonstrate that prediction error signaling is reflected in human LFPs.

Recent work by Jahn et al. (2014) and Silvetti et al. (2014) suggest that the dACC signals both predictions and errors related to those predictions, but that these two signals may be localized to distinct zones of the dACC. Our results suggest a degree of lateralization across hemispheres in the dACC response to outcomes. Right dACC is involved in the initial formation of predictions while left dACC does not signal prediction error *per se*, but a fundamental dynamic signature of the rule-updating process as suggested by the late components in Figure 3, agreeing with fMRI work by O’Reilly et al. (2013) showing that dACC activity updates future behavior. The timing and overall response profile of these components were generally consistent with those reported recently in human electroencephalography (EEG) studies using a similar reward-learning task (Philiastides et al., 2010; Fouragnan et al., 2015, 2017). In those studies, the early component was shown to represent a quick evaluation of the outcome along a good/bad axis, whereas the later component was more directly involved in updating/learning stimulus-reward associations. Both our multivariate analysis (Figure 4) and our ERSP analysis (Figure 5C) show evidence that the early and late components are temporally separated by a single theta oscillation and are in reaction to negative outcome valency, lending credence to studies that suggest that predictions and rule adjustment following feedback are primarily processed through activity in the theta frequency band (Klimesch, 1999; Womelsdorf et al., 2010; Cavanagh and Frank, 2014). Individual, separate components were observed following correct feedback in the left dACC (Figure 5C), implying less of a need for rule adjustments following expected responses as seen by RT results (Jensen and Tesche, 2002).

Do our findings offer any clarification on the dACC’s role in foraging theory (Krebs et al., 1977; Stephens and Krebs, 1986)? The IED task that our dACC subjects performed is a cognitive task with clear foraging parallels—how do organisms make decisions and evaluate the potential consequences of choices as they arise? In our case, the IED task placed subjects into an evolving environment, where rewards (positive feedback and successfully progressing through the task) were fully known but the consequences (the rules) of a particular choice were not fixed and had to be reassessed and at times relearned as the task progressed (Walton and Mars, 2007). Such a scenario is useful as it allowed us to control and manipulate the parameters of the task through varying stimulus-response-outcome combinations in order to introduce elements of unfamiliarity and expectancy. Our results revealed the dACC to be involved throughout the decision-making process, both before decisions are made at the time of stimuli presentation and post-decision after feedback is delivered. The right hemisphere dACC was shown to respond to the introduction of stimuli while the left hemisphere dACC was shown to be sensitive to two distinct variables: the receipt of feedback and the further recognition and processing of error. Our results are consistent with the idea that the dACC is implicated in linking actions with outcomes and using changes in feedback to provoke the updating of future decision-making paradigms (Botvinick et al., 2001; Hayden et al., 2011; Blanchard and Hayden, 2014). Essentially, we offer clarifying evidence of lateralized decision-making in line with foraging theories in the dACC. When designing studies for humans or non-human primates, however, one should be cognizant that in real foraging situations outcomes are seldom composed of simple categorically correct or incorrect choices (Walton and Mars, 2007). This, in effect, limits the sort of conclusions that can be drawn from results. Future studies of dACC executive function would benefit from designing more “natural, stochastic, experimental designs to better capture diverse types of foraging-related decision-making that the dACC and the other subdivisions of the PFC evolved to address in humans and other mammals.

Our findings are relevant to evaluating competing schools of thought regarding dACC function. Our experimental results put us in the camp of Kolling et al. (2016b) wherein the dACC is thought to play a leading role in the regulation of behavioral adaptation and persistence. Their theory suggests the influence of decision-making factors such as difficulty or conflict are secondary to and derived from the dACC’s role in evaluating behavioral change. Expected value-related, outcome-related, and model updating-related activity in the dACC all work together to regulate behavioral adaptation in the face of updating environments and stimuli. We observed dACC activity in response to novelty during stimuli presentation and following negative feedback; events and subsequent activity that allow the dACC to adjust the behavior of our subjects and mirror the behavioral updating of Kolling et al. (2016b). In contrast, while Shenhav et al.’s Expected Value of Control theory highlights dACC’s role specifying the optimal allocation of control, they propose that dACC signal strength varies in a graded fashion depending on the benefit or effort required in decision making, which was not observed in our subjects (Shenhav et al., 2013, 2016). In all fairness, the IED task is not an ideal paradigm for truly teasing apart these theories, between which there is a remarkable degree of agreement. Both theories highlight the dACC’s role in signaling the value of behavioral paradigms to set up future behavior. These theories do have different implications for the role of the dACC in the grander scheme of cognition and executive function: is the dACC a controller separate from the workings of cognition that prompts sensorimotor behavior (Shenhav et al., 2016) or is it an integrated part of a circuit subject itself to outside factors (Kolling et al., 2016b)? While our work does not necessarily prompt stronger certainty in this debate, it does provide rare intracortical electrophysiological recordings that may lead to a greater understanding of the dACC and its roles in executive function.

### dACC Laterality

This study reports findings from three subjects with bilateral dACC implantation of DBS electrodes. The landmark used to target the dACC was the tip of the frontal horn of the lateral ventricle; a target highly subject to significant interindividual variability (Boccard et al., 2016). Our MRI data (Figures 1A,B) indicate that our electrodes were localized to the posterior end of the rostral cingulate zone (Picard and Strick, 1996; Ridderinkhof et al., 2004; Amiez et al., 2013). Our surgical procedure assures that our electrodes are inserted symmetrically. However, there it is no guarantee that two symmetric coordinates on the medial walls must be comparable. Many recent studies have shown that the cingulate cortex has a high rate of interindividual variation in terms of morphology and functional organization, being heterogeneous along the dorso-ventral and rostro-caudal axes (Vogt et al., 1995; Amiez et al., 2013; Scholl et al., 2017). To justify exploring dACC functional localization and laterality and to avoid the chance that effects could be explained by simple intersubject cortex variability, we performed DTI analysis. DTI is a technique that quantifies the anisotropy of water diffusion in the brain and allows for the tracing of white matter connectivity of tissue adjacent to dACC electrode contacts (Basser et al., 1994). In our subjects, the strongest connectivity at the electrode location was found to be the left hemisphere’s SMA, with more modest connectivity found with the right hemisphere’s SFG and the SMFG (Figures 1C,D). These DTI observations were consistent with known human and non-human primate dACC anatomical connectivity with regions of the frontal cortex and motor areas and confirmed that electrode placement was bilaterally in the dorsal part of the ACC (Koski and Paus, 2000; Asemi et al., 2015; Neubert et al., 2015). Further, as connectivity was similar across hemispheres, we can make claims that our symmetrically implanted electrodes are capturing signals from comparable (in a connectome sense) regions of the dACC. Therefore, our MRI and DTI results assure that we can probe questions of functional subdivisions and laterality in the dACC.

Functional subdivisions of ACC have been proposed before, with the evidence of intersubject variability allowing for additional discrepancies (Polli et al., 2005; Taylor et al., 2006; Lutcke and Frahm, 2008). While lateralization is not often reported in most PFC studies, some groups have observed cingulate executive function processing specialization in either the left or the right hemisphere (Konishi et al., 1998; Garavan et al., 1999, 2003; Menon et al., 2001; Rubia et al., 2001; Taylor et al., 2006). Our results are in agreement with these previous studies that investigated hemispheric lateralization of executive function in the dACC. We were able to show that individual, separate components could be observed following the receipt of incorrect feedback in the left hemisphere dACC. This may indicate that both early and late components would be expected to be different between the two hemispheres only if one compared a correct guess at a familiar set of objects vs. an incorrect guess at a novel set of objects. The presence of theta and high delta frequency activity in the left and right dACC during the different phases of the task and theta’s perceived involvement in a multitude of cognitive processes including decision making, outcome valence, reaction to novelty, and recalculation of predictions in medial frontal regions lend further credence to this theory (Klimesch, 1999; Jensen and Tesche, 2002; Lindsen et al., 2010; Womelsdorf et al., 2010; Cavanagh and Frank, 2014). Unfortunately, we can only make limited statements regarding hemisphere dominance in this study as we had no left-handed subjects. While there is precedence of discrete dACC lateralization of function with respect to verbal and figural fluency, the present study presents the first evidence of dACC lateralization during executive function (Geisseler et al., 2016).

### Limitations

This study examines the electrophysiology underlying executive function in the dACC of chronic pain subjects. Could the neurological condition of our subjects influence the results of our study in any way? Pain has been shown to influence neurophysiological test performance, including both attentional and executive functions (Eccleston, 1995; Grisart and Plaghki, 1999; Nicholson et al., 2001; Moriarty et al., 2011). However, the precise nature of abnormal performance effects on tasks of executive function in chronic pain patients is unclear and controversial. When designing a study of cognitive ability in pain subjects, one factor that should be accounted for is the influence of psychomotor speed on the chosen cognitive task (Oosterman et al., 2012). Psychomotor abilities relate to the relationship between cognitive functions and physical movements such as the ability to detect and respond to rapid changes in the environment (Lezak, 2004). Significantly, psychomotor slowing has been a consistent finding in chronic pain patients, and at least part of the reported declines in executive function reflect this slowing (Hart et al., 2000; Lezak, 2004; Oosterman et al., 2012). This is particularly crucial in cognitive testing as some of the most commonly used tests of executive function, such as the Stroop Test, are strongly dependent on psychomotor speed ability (Lemelin and Baruch, 1998). We chose the IED task as a neuropsychological measure, in part, as it is not affected by psychomotor speed and so avoid the possibility of compromised basic cognitive processes (Sahakian and Owen, 1992). To further avoid potential cognitive deficits, patients referred to our single-center team for chronic pain DBS treatment were screened with a neuropsychological evaluation that excluded psychiatric disorders and ensured minimal cognitive impairment (Boccard et al., 2013, 2014).

## Conclusion

These results should prove to be a useful addition to the body of dACC literature that provide insight into the dACC’s role in executive function. To our knowledge, this study represents the first bilateral dACC electrode recording study from awake humans performing a cognitive task with sensory cue, motor action, and sensory feedback components. Localized LFP recordings from the ACC in humans are rare, and this study contributes unique electrophysiological measurements with high spatial and temporal resolution not obtainable via fMRI or EEG methods. Collectively, our results indicate that the human dACC exhibits theta frequency band event-related LFPs throughout the course of a cognitive task of executive function. The right hemisphere dACC was active during the presentation of sensory stimuli, when subjects began to formulate predictions of behavioral outcomes. The left hemisphere dACC exhibited two spatiotemporally separated signals related to processing and responding to behavioral feedback—an early signal tracking outcome valence and a late signal related to prediction error. Our laterality findings are further established through the inclusion of post-operative CT and DTI analyses that confirm our electrode placement in the dACC and connectivity uniformity between hemispheres. Essentially, the human dACC is active in a lateralized manner during decision-making and afterwards when outcomes are processed, prompting behavioral adaptation.

## Author Contributions

AW, TA, MG and AG: conceptualization and methodology. AW, MP, SB, MA, MW, TA, MG and AG: formal analysis. AW, SB, JF, TA, MG and AG: investigation. TA, MG, JF and AG: resources. AW, SB, MG and MP: data curation. AW, MA, MG and AG: writing—original draft. AW, MA, MP, MG, TA, MW and AG: writing—review and editing. AW, MG, SB and MP: visualization. MG, TA and AG: supervision. MG, JF, TA and AG: funding acquisition.

## Conflict of Interest Statement

The authors declare that the research was conducted in the absence of any commercial or financial relationships that could be construed as a potential conflict of interest.
